# Forced Swim Reliability for Exercise Testing in Rats by a Tethered Swimming Apparatus

**DOI:** 10.3389/fphys.2018.01839

**Published:** 2018-12-19

**Authors:** Ivan G. M. dos Reis, Luiz E. B. Martins, Gustavo G. de Araujo, Claudio A. Gobatto

**Affiliations:** ^1^Laboratory of Applied Sport Physiology, School of Applied Sciences, University of Campinas, Limeira, Brazil; ^2^School of Physical Education, University of Campinas, Campinas, Brazil

**Keywords:** exercise physiology, exercise testing, exercise prescription, anaerobic threshold, aerobic capacity

## Abstract

To assess the physical capacity of rats in forced swim tests, the animal should perform a continuous activity (CON) at the surface to avoid apnea. Bobbing movement (BOB), vigorous paddling known as climbing (CLI), and diving activity (DIV) are inadequate swimming patterns known to increase the exercise intensity variability, impairing the test reliability. Thus, the exercise work accomplished and related physiological variables, such as the blood lactate concentration, may be unreproducible in forced swim. This study aimed to verify the exercise work reproducibility in rats with a 30-min test–retest at maximal lactate steady state (MLSS) intensity using a tethered-swimming apparatus that analyzes swimming patterns by the direct measurement of swimming force. Additionally, it was determined the swimming force and duration of CON, BOB, CLI, and DIV at physiologically different exercise-intensities. The swimming force at MLSS (*n* = 64) was 38 ± 7 g*f*.Kg^-1^, while the blood lactate concentration was 4.2 ± 1.6 mmol.L^-1^. In the test–retest (*N* = 23), swimming force (36.6 ± 7 g*f*.Kg^-1^ vs. 36.4 ± 7 g*f*.Kg^-1^) and blood lactate concentration (4.7 ± 1.7 mmol.L^-1^ vs. 4.2 ± 1.7 mmol.l^-1^) were similar, but only the swimming force was highly correlated (0.90 and 0.31). Although it was not statistically different, the swimming force for CON tends to be slightly lower than CLI and slightly higher than BOB independently of exercise-intensity. The CON pattern predominates (∼52.8 ± 18%) at intensities below and of MLSS but BOB was the swimming pattern more often observed above MLSS-intensity (52.6 ± 18%). The present study used a tethered swimming apparatus to investigate the reliability of forced swim tests for exercise testing in rats and better understand the swimming patterns when determining the MLSS, but the results can be extended to any study that rely on forced swim for exercise testing and training. The result suggests that, at least at intensities of physiological stability, the exercise work accomplished by rats is reproducible in forced swim, but the blood lactate concentration seems to be affected by other factors, such as the apnea and stress caused by the possibility of drowning, besides the exercise-intensity.

## Introduction

Is the exercise work accomplished by rats in forced swim tests reproducible? Although the forced swim has been widely used in studies for exercise testing and training in rats, to the best of our knowledge, the exercise work reproducibility was never verified. Employed as an exercise modality for animals due to many known advantages over other modalities, the forced swim has specific disadvantages as the difficulty to control the exercise intensity (Dawson and Horwath, 1970; Kregel et al., 2006).

Although the exercise intensity in forced swim tests can be indirectly graded by the addition of weight load, attached to the body (Gobatto et al., 2001; de Araujo et al., 2007) or tail (Kregel et al., 2006), to decrease the time until exhaustion (McArdle and Montoye, 1966), a rat must exercise uniformly and continuously (CON) at the surface with a minimum time submersed to perform this type of physical activity appropriately. However, this swimming pattern is not always observed. Instead, some rats can resort to non-continuous activity that may be constructed as escape or survival strategy to avoid the stressful possibility of drowning (Kregel et al., 2006).

In shallow tanks (50 cm deep or shallower), a typical strategy of swimming rats is resting while they sink toward the bottom and, when they reach the bottom, push off for return to the surface (Kregel et al., 2006). Deeper tanks can inhibit this swimming pattern (Kregel et al., 2006), but in this condition the rats may bob (BOB) only near the surface without reaching the bottom. This swimming pattern is mainly observed when exhaustion is imminent and the animal needs to be removed from the tank to avoid drowning due to fatigue established. Other inadequate swimming pattern is the excess of paddling or propulsion known as climbing (CLI), commonly seen in the first minutes of activity. It is likely that BOB and also diving activity (DIV), when the rat explores the tank bottom for trying to escape, may impair the swimming performance by causing hypoxia due to intermittent periods of apnea (Kregel et al., 2006). In turn, a swimming pattern as CLI could lead to precocious fatigue and exhaustion (Kregel et al., 2006).

There is a growing concern in establishing standards to investigate physiological responses in animals (Booth et al., 2009), and the wide use of forced swim without attention to its procedures is worrisome. Previous studies have investigated and proposed solutions for several forced swim issues (Scheer et al., 1947; McArdle and Montoye, 1966; Dawson and Horwath, 1970; Kramer et al., 1993; dos Reis et al., 2011a; Hohl et al., 2011; Lima et al., 2017), but none has addressed the swimming patterns specifically. Only by observation, it is possible to realize that rats can behave distinctively during forced swim tests. Once the inadequate swimming patterns are sources of exercise intensity variability, the bias it can introduce to the exercise work accomplished is still unknown.

Also, several studies have standardized protocols of forced swim for exercise testing and preconized the individual assessment of physical capacity in rats (Gobatto et al., 2001; de Araujo et al., 2007; dos Reis et al., 2011a). In this way, Gobatto et al. (2001) have succeeded in determining the maximal lactate steady state (MLSS) in rats submitted to swimming. The MLSS has important implications for the study of sports and health sciences because it represents the boundary or highest effort intensity of lactate stability. Therefore, the blood lactate appearance equals removal at MLSS-intensity but above it the concentration increases until exhaustion (Heck et al., 1985; Gobatto et al., 2001; Billat et al., 2003).

The mean value of swimming force performed during a BOB pattern would be lower than CON due to its stop and go movement characteristic. Thus, the difficult to sustain a CON pattern would be higher than BOB and at high-intensity exercise the BOB pattern would be more frequently observed. Moreover, when the activity can be maintained for a long period of time as it is at MLSS-intensity, it is expected a predominance of CON pattern. Instead, at intensity above MLSS, the occurrence of inadequate BOB pattern would increase, for saving energy, due to the fatigue accumulated in a short period of activity. Additionally, the reproducibility of blood lactate concentration, as a physiological parameter, may be impaired in this condition due to also being affected by other factors besides the exercise intensity, such as the apnea and stress caused by the possibility of drowning.

As pointed out above, attesting the reproducibility of mechanical and physiological parameters and the appreciation of swimming patterns is strictly important to improve the forced swim reliability for exercise testing in rats, as well to reduce the number of animals required to observe significant treatment effects. However, the literature lacks information about the swimming force profile in rats or whether it may change in function of the exercise intensity. Previously, a tethered-swimming apparatus for rats was developed (dos Reis et al., 2011b) and used to analyze the swimming patterns by recording direct measurements of swimming force. Thus, the aim of the present study was (1) to verify the exercise work reproducibility in rats with a 30-min test–retest at the MLSS-intensity using a tethered swimming apparatus. Additionally, by means of the swimming force analysis, it was determined (2) the swimming force and duration of CON, BOB, CLI, and DIV at physiologically different exercise-intensities.

The results of the present study would collaborate with the legacy of studies that already investigated forced swim issues toward a more accurate access of exercise capacity in rats. The novelty is the employment of the tethered swimming apparatus bringing unprecedented information regarding the swimming force profile along the swimming session in different exercise intensities. Such data shed new light on the reproducibility and limitations of the use of forced swim in exercise testing and training studies in the field of exercise physiology. Additionally, it can guide other researchers for the adequate conduction of experiments involving forced swim (e.g., the identification of swimming patterns or conditions that may impair the physiological assessment).

## Materials and Methods

### Ethics Statement

Prior the experiment, all procedures were approved by the Ethics Commission in the Use of Animals (CEUA) at the University of Campinas (UNICAMP), Brazil (protocol number: 2426-1). Additionally, procedures were in line with the ethical principles of animal experimentation adopted by the Brazilian Society of Science in Laboratory Animals (SPCAL), current legislation (law number: 11.794, October 8, 2008; decree number: 6899, July 15, 2009).

### Animal Care

Male Wistar rats, with 120 days old at the beginning of the experiment, obtained from Multidisciplinary Nucleus for Biological Investigation in Laboratory Animals from the University of Campinas (Campinas, São Paulo, Brazil), were housed in an air-conditioned room (21 ± 2°C), with 12–12 h light-dark cycle (6 am to 18 pm), into polyethylene boxes (49 cm length × 34 cm width × 16 cm height) with five animals each. They had free access to commercial rodent food (Nuvilab, Parana, BRA) and water.

It seems that the familiarization to the water environment reduces fatigue time differences among rats in forced swim tests with constant workloads (McArdle and Montoye, 1966). Thus, all animals swam 5 min per day during 15 out of 21 days. The familiarization entailed swimming in shallow water, with daily increments in depth until the animals were unable to stand on the tank floor.

### Tethered-Swimming Apparatus

**FIGURE 1:**
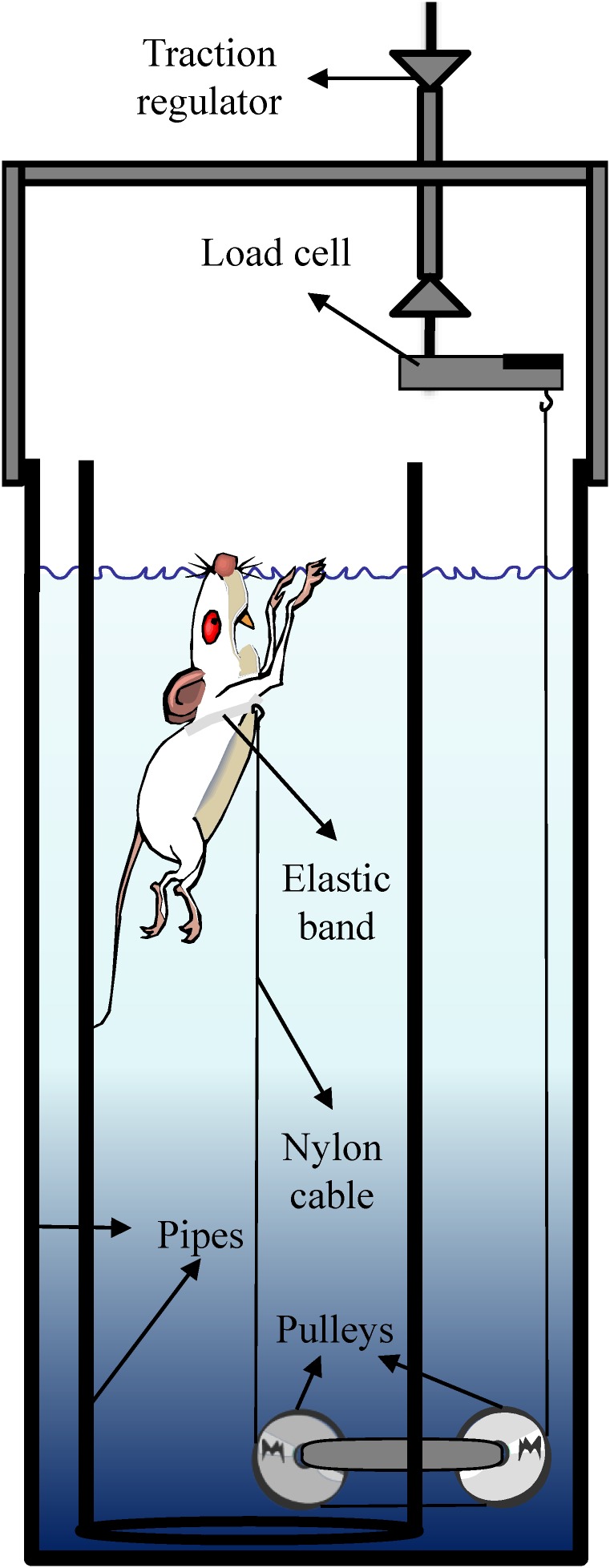
Tethered swimming apparatus.

The tethered-swimming apparatus comprises a cylindrical pipe of polyvinyl chloride (20 cm inside diameter × 100 cm deep) located inside a water tank (30 cm inside diameter × 100 cm deep). The swimming took place in 80 cm deep warmed water (34 ± 1°C) (Harri and Kuusela, 1986). To avoid the effects of crowding on performance (Wilber, 1959; Wilber and Hunn, 1960), the rats swam individually while pulling vertically a nylon cable attached around their chest by an elastic band so that their movements were not restricted (dos Reis et al., 2011b). The nylon wire (∼180 cm) was leaded horizontally from the inside toward the outside of the pipe by a pulley centrally positioned at the bottom, while an outside pulley, disposed in series, leads the other extremity of the nylon cable vertically to a load cell (Figure 1).

The data acquisition system comprises a MKPW-2 load cell (MK Control and Instrumentation, São Paulo, Brazil), MKTC5 signal amplifier (MK Control and Instrumentation, São Paulo, Brazil), USP-6008 signal conditioning module (National Instruments, Austin, TX, United States), and LabView Signal Express 2009 software (National Instruments, Austin, TX, United States).

The system was calibrated by replacing the rat by an analog dynamometer of 50 g with 1 g of precision. During the tests, the swimming force can be assessed in real time, and at the early seconds of the test it is manually graduated with a precision of ∼3–4 g, due to the swimming force oscillation, by the vertical displacement of the load cell fixed to a support.

### Maximal Lactate Steady State Tests

For an experienced evaluator, it is easy to determine when submersion is due to exhaustion or a diving reflex (Miller and Darrow, 1941). Thus, exhaustion was assumed only when a rat was trying, but was unable to reach the surface over 10 consecutive seconds (Scheer et al., 1947; McArdle and Montoye, 1966).

In the adapted MLSS protocol developed with the tethered swimming apparatus, each animal underwent 3–4 attempts to swim for 30 min at constant exercise intensity, with each taking place at approximately the same time of day (between 8 am and 18 pm), with a 48-h interval and at different intensities, of ∼10–35 g force (g*f*). Blood samples of 25 μL were collected for lactate measurement from a distal cut in the animal’s tail at rest, and at 10th and 30th minute of exercise. The MLSS intensity was the highest workload in which blood lactate concentration varied less than 1 mmol.L^-1^ between the 10th and the 30th minute of exercise (Gobatto et al., 2001; Beneke et al., 2003). The first trial was carried out at effort intensity between the range of ∼20–24 g*f*, while the next trial were carried out at higher (+4 g*f*) or lower (-4 g*f*) intensity in case of blood lactate variation above or below 1 mmol.L^-1^, respectively.

### Blood Sampling and Lactate Determination

Blood samples were collected using heparinized capillary tubes and transferred to 1.5 mL microcentrifuge tubes containing 400 μL of trichloroacetic acid [4%] (C_2_HCl_3_O_2_). After being centrifuged for 3 min, 50 μl of the supernatant was placed into tubes containing 250 μl of a solution of glycine/EDTA, hydrazine hydrate 88% (pH 8.85), lactate dehydrogenase from bovine heart (L3916, Sigma-Aldrich, St. Louis, MO, United States), and β-nicotinamide adenine dinucleotide hydrate (N7004, Sigma-Aldrich, St. Louis, MO, United States), as previously described (Engel and Jones, 1978). Following incubation for 20 min at 37°C, absorbance was determined at 340 nm using a microplate reader (ASYS Expert Plus UV, Biochrom, Cambridge, United Kingdom) and normalized with a calibration curve.

### Swimming Patterns

Signal sampling rate was set to 200 Hz to oversample the swimming force and prevent aliasing. Digital signal processing and data statistical treatment were made in MatLab^®^ 7.0 (MathWorks^TM^) software. After verified by a fast fourier transform that the power of swimming force signal is below 5 Hz (data not presented), the raw data was processed by a 4th order butterworth and 5 Hz lowpass digital filter to remove the spikes of high frequency noise.

As described below, the swimming patterns was determined for each swimming force signal sample according with its own value in grams force (g*f*) and by the average of swimming force variation (difference between consecutive signal samples) of the three hundred previous and forward samples in grams force by seconds (g*f*. s^-1^), like a moving average of the swimming force variation in a 3-s interval (601 samples). Thus, a swimming force signal sample was classified as BOB swimming pattern when it was equal or higher than 3 g*f* and the mean variation around it was higher than 20 g*f*.s^-1^. Also, the swimming force signal sample was classified as BOB swimming pattern when it was lower than 3 g*f* and the mean variation around it was equal or higher than 10 g*f*.s^-1^ (Table 1). In turn, a swimming force signal sample was classified as CLI swimming pattern when it was equal or higher than 3 g*f* and the mean variation around it was equal and lower than 5 g*f*.s^-1^ (Table 1). The swimming force signal sample was classified as CON swimming pattern when it was equal or higher than 3 g*f* and the mean variation around it was higher than 5 and equal or lower than 20 g*f*.s^-1^ (Table 1). Finally, the SF signal sample was classified as DIV swimming pattern when it was lower than 3 g*f* and the mean variation around it was lower than 10 g*f*.s^-1^ (Table 1).

**Table 1:** Mean values of swimming force signal variation (g*f*.s^-1^) from a 601 samples interval used as criteria to classify the central sample as bobbing, climbing, continuous, or diving swimming patterns.

	Bobbing	Climbing	Continuous	Diving
Mean variation (g*f*.s^-1^)	Higher than 20^∗^; or equal or higher than 10^#^	Equal or lower than 5^∗^	Higher than 5 and equal or lower than 20^∗^	Lower than 10^#^

*^∗^Only values equal or higher than 3 gf; ^#^Only values lower than 3 gf.*

**FIGURE 2:**
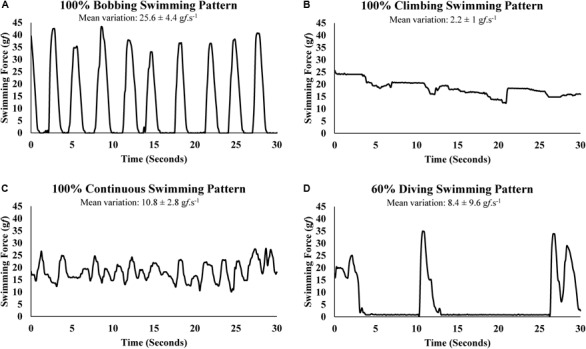
Swimming force variation (g*f*.s^-1^) of swimming patterns: **(A)** bobbing, **(B)** climbing, **(C)** continuous, **(D)** diving.

The direct determination of the SF enabled the graphic representation of swimming patters, which were classified according to the criteria established in Table 1. Well-characterized sections of swimming force representing each swimming pattern are shown in Figure 2.

### Statistical Analysis

Data is presented as mean ± standard deviation. The mean swimming force for each animal was determined by averaging the swimming force signal samples of the total length of test, and the coefficient of variation is equivalent to the averaged ratio between the standard deviation and mean of swimming force samples. All variables had normal distribution (Lilliefors test), and the variance among effort intensities (below, equal, and above the MLSS) were analyzed by ANOVA Factorial, when necessary followed by a pair-wise multiple comparison method between groups. The *t*-test for dependent samples and intraclass correlation (absolute agreement) was used for test and retest comparison. Bland–Altman plots (Bland and Altman, 1999) was used to verify the agreement between the values of test and retest. The significance was always set at *p* < 0.05.

## Results

Due to intercurrences, it was not possible determine the MLSS for sixteen animals which blood lactate concentration was inconsistent among exercise intensities or was stable or unstable at all tests or were incapable, for avoiding its drowning, of finishing enough number of tests. Thus, the protocol proposed in this study was succeed in determining the MLSS of sixty-four out of eighty animals (success rate of ∼80%), which 23 were used in the reproducibility tests. The blood lactate concentration at 10th and 30th minute of test were different among effort intensities (Table 2).

**Table 2:** Mean values of blood lactate concentrations (BLC) at intensities below, equivalent, and above the MLSS at rest, 10th, and 30th minute of exercise, of 64 rats (except the intensity above MLSS).

*N* = 64	Rest (mmol.l^-1^)	10th (mmol.l^-1^)	30th (mmol.l^-1^)
Below	1.3 ± 0.5	3.5 ± 1.3	3.1 ± 1.3
MLSS	1.3 ± 0.5	4.3 ± 1.5^∗^	4 ± 1.7^∗^
Above	1.5 ± 0.4	6.3 ± 2.1^∗#^	–

*Different of ^∗^Below and ^#^MLSS.*

As expected, the mean swimming force was different between intensities below, equivalent, and above the MLSS (Table 3). However, the mean coefficient of variation was not affected by the exercise intensity (Table 3). When analyzing the swimming patterns, at intensity below MLSS the swimming force for BOB was slightly lower than CLI and CON and a tendency is observed at intensities above and of MLSS (Table 3). The swimming pattern most often observed at intensities below and of MLSS is CON, followed by BOB and CLI, while DIV represents only a very small amount of time spent in forced swim (Table 3). At intensity above MLSS the BOB pattern predominates (Table 3).

**Table 3:** Mean values of swimming force (SF), coefficient of variation (CV), and percentage of time spent in each swimming pattern (total time) for bobbing (BOB), climbing (CLI), continuous (CON), diving (DIV) at intensities below, equivalent, and above the MLSS, of 64 rats.

*N* = 64	Swimming Force (g*f*.k^-1^)			Coefficient of Variation (%)			Total Time (%)		
	Below	MLSS	Above	Below	MLSS	Above	Below	MLSS	Above
BOB	29 ± 9^b^	35 ± 9^ab^	40 ± 8^a^	71 ± 26^b^	59 ± 16^c^	56 ± 19^ac^	33 ± 25^a^	37 ± 22^a^	53 ± 18^b^
CLI	38 ± 10^a^	43 ± 9^a^	52 ± 12^c^	15 ± 7^d^	13 ± 6^d^	11 ± 6^d^	11 ± 15^c^	11 ± 15^c^	7 ± 11^c^
CON	35 ± 7^a^	40 ± 7^a^	48 ± 6^ac^	29 ± 6^a^	26 ± 6^a^	24 ± 6^ad^	54 ± 20^b^	52 ± 17^b^	39 ± 13^a^
DIV	–	–	–	–	–	–	1 ± 2^c^	1 ± 2^c^	1 ± 2^c^
Mean	33 ± 7	38 ± 7^#^	44 ± 6^#*^	43 ± 14	40 ± 12	43 ± 11	–	–	–

*Different letters represent significant differences; Different of ^#^Below and ^∗^MLSS.*

In a test and retest at the MLSS, the swimming force was reproducible and highly correlated, but, besides similar mean values, the blood lactate concentration was poor correlated (Table 4). The swimming force on different swimming patterns were similar between test and retest but the total time of each swimming patterns had a large variation except for CON (Table 4).

**Table 4:** Mean values of swimming force (SF), percentage of time spent in each swimming pattern (total time) for bobbing (BOB), climbing (CLI), continuous (CON), diving (DIV), and blood lactate concentration (BLC) followed by the *P*-value of test t (*P*-value), coefficients of variation (CV), effect size (ES), and intraclass correlation (ICC) between test (SF1 and BLC1) and retest (SF2 and BLC2), of 23 rats.

*N* = 23	SF1 (g*f*.k^-1^)	SF2 (g*f*.k^-1^)	Total time 1 (%)	Total Time 2 (%)
BOB	33.6 ± 10	32 ± 9	39.3 ± 19	35.4 ± 23
CLI	40.1 ± 11	41.5 ± 11	6.7 ± 11	12.2 ± 14
CON	38.9 ± 7	38.6 ± 7	53 ± 16	51 ± 17
DIV	–	–	0.9 ± 1	0.86 ± 1

	**SF1 (g*f*.k^-1^)**	**SF2 (g*f*.k^-1^)**	**BLC1 (mmol.l^-1^)**	**BLC2 (mmol.l^-1^)**

Mean	36.4 ± 7	36.6 ± 7	4.4 ± 1.6	3.9 ± 1.3
*P*-value	0.94		0.17	
CV (%)	6.7		35	
ES	0.04		0.35	
ICC	0.90^∗^		0.31	

*^∗^Significance between test and retest.*

**FIGURE 3:**
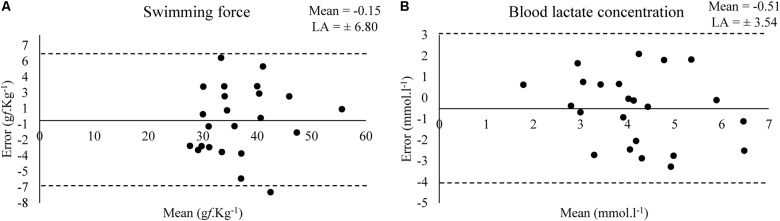
Bland–Altman plots for the values of swimming force **(A)** and **(B)** blood lactate concentration in the test and retest at MLSS-intensity (*N* = 23).

Bland–Altman plots shows the estimated bias and limit of agreement (LA) of swimming force and blood lactate concentration between a test and retest at MLSS (Figure 3).

## Discussion

The present study used a tethered swimming apparatus to investigate the reliability of forced swim tests for exercise testing in rats and better understand the swimming patterns when determining the MLSS, but the results can be extended for other studies that rely on forced swim tests for exercise testing and training. More important than the moment in which each swimming pattern takes place in the swimming session, is how long it last and how much it can affect the amount of exercise work done by the animal. When added together, all the inadequate swimming pattern (BOB, CLI, and DIV) accounted for almost ∼50% of the amount of time in forced swim at intensities bellow or equivalent to MLSS, but the CON pattern still predominates (Table 3). Thus, the swimming force was similar between test–retest (Table 4), which indicates that the amount of work accomplished was reproducible at MLSS-intensity. However, at intensity above MLSS-intensity, the BOB pattern was the most often swimming pattern (Table 3) and, at this condition, the swimming performance may not be reproducible. Such assumption still needs to be confirmed.

Although the small effect size (0.35), acceptable agreement and not higher bias (-0.54 mmol.l^-1^) observed in the Bland–Altman plot (Figure 3) in relation to the mean value (∼4 mmol.l^-1^), the blood lactate concentration was low correlated (0.31) in a test–retest (Table 2) (Hopkins et al., 2009). Abel (1994b) shown that the serum lactate response occurred to a higher extent in unloaded water immersion compared to treadmill running or foot shock, although without equalizing the level of physical activity among the conditions, and suggested that the pattern of physiological changes in response to forced swim happens not only due to physical activity and stress-related trauma. Therefore, considering the lactate multifactorial responsiveness in this condition, the poor correlation between blood lactate concentration values of test–retest was an expected result. However, blood lactate concentration means were similar between test–retest and the exercise work accomplished was not affected (Table 4). Moreover, Gobatto et al. (2001) shown that the rat blood lactate concentration response to forced swim at constant workload is similar to the response found in exercising humans and can be used for MLSS determination. Thus, MLSS-blood lactate concentration utilization as a parameter provides an individualized determination of exercise intensity in rats due to be ensured by a physiological phenomenon, but researchers should have caution in using it (i.e., animals with inconsistent blood lactate concentration among exercise intensities should be excluded from the sample) as some bias may be present.

A limitation of the present study regarding the swimming patterns analyses is the lack of footage to synchronize the swimming force with visual information and, therefore, the swimming patterns at given force were presumed. However, while a visual analysis would provide a subjective interpretation, the present study proposed an objective and, due to the direct measure of swimming force, reliable method to classify the swimming patterns by the swimming force variation (Figure 2).

The interpretation of swimming pattern in forced swim has been used as animal model for the study of depression (Abel, 1994a) and is a valuable tool to evaluate the relationship between stress and depression, as well as their physiological response patterns (Abel, 1993). Specifically, the time of immobility of a testing animal is associated to a state of behavioral despair in response to stress (Abel, 1993). This immobility is not observed in forced swim tests carried out with load weight (Gobatto et al., 2001) that forces the animals to swim for remaining at the surface. However, rats are incapable of maintaining a constant swimming force (Kniazuk and Molitor, 1944) and, for the first time, the swimming force variability was determined (Table 3).

When submerged in deeper water, rats may behave individually different, including vigorous paddling, increased diving, and physiological reactions (e.g., hyperlactatemia, hypocarbia) that may or may not also occur when rats are tested at more traditional shallower depths (Abel, 1994a). There are several mentions of swimming patterns in the literature (Miller and Darrow, 1941; Kniazuk and Molitor, 1944; Abel, 1994a; Kregel et al., 2006) and its descriptions seems to be similar to BOB, CLI, and DIV patterns observed in the MLSS tests. Unfortunately, some swimming patterns may impair metabolic assessments by introducing intermittent bouts of apnea. Apparently, these swimming patterns are survival or escape strategies and could not be totally avoided. Perhaps, new strategies of familiarization to the water and swimming exercise could reduce the incidence of inappropriate swimming patterns, such as BOB, CLI, and DIV.

It was previously reported that rats seem to try to conserve their energy by taking a rest at frequent intervals, by sinking to the bottom of the tank (Kniazuk and Molitor, 1944). This description is similar to the swimming pattern named here as BOB in reference to the stop and go or intermittent activity that some animals adopt, mainly when the exhaustion is eminent, as swimming strategy for saving energy.

To prevent the BOB pattern in swimming rats (Kregel et al., 2006), it was suggested water tanks of at least 100 cm deep (McArdle and Montoye, 1966) and to add weights to the base of the tails (Flaim et al., 1979). Indeed, during swimming with weights (information from other studies of our group) and tethered swimming, an 80 cm water column was deep enough to prevent rat voluntary sinking until reach the bottom to push toward the surface. However, instead BOB until reach the bottom, some rats may do a shorter BOB by sinking just enough to rest a few seconds before have to swim vigorously for return to the surface (Figure 2A).

The swimming force analysis showed that BOB pattern has lower mean value (not statistical) and higher CV than CON pattern independently of the intensity (Table 3). Moreover, BOB pattern accounts to ∼35% of time at the intensities bellow and equivalent to MLSS and it is the predominant SP at the intensity above MLSS (Table 3). Therefore, BOB seems to reduce the work performed due to the inconstant activity (Figure 2A) and, in exchange, the animal undergoes brief intervals of apnea. Therefore, the tethered swimming provides a more reliable determination of work than the weight load attached to the tail or body, mainly at the intensities above MLSS when a rat commonly resorts to BOB pattern.

Kniazuk and Molitor (1944) described a swimming pattern that resembles CLI pattern, it seems to affect stressed rats, which exhausted their energy rapidly and prematurely. In another swimming pattern description similar to a CLI pattern, Abel (1994a,b) reported physiological responses, such as hyperlactatemia, associated with vigorous paddling behavior provoked by the submersion of rats in a cylinder of water too deep to they keep their heads above water by standing on their feet. This swimming pattern would stop eventually not because of exhaustion but because the rats learn to adapt to an inescapable test situation and serum lactate declines precipitously after that (Abel, 1994a,b) Occasionally, mainly in the early minutes of the MLSS test, some animals exceed greatly the swimming force needed to remain at the surface (Figure 2B). Indeed, the higher blood lactate concentration value (non-significant) at 10th minute of exercise compared with 30th minute (Table 2) may be due the initial stress of water exposition. Generally, the animal tends to decrease paddling after few seconds or minutes. However, it is possible that even a short period of CLI pattern may promote or anticipate fatigue.

To reduce the CLI pattern, it was suggested round tanks with large surface area and a fair amount of space between the water level and the top of the tank (Kregel et al., 2006). Previously, it was showed a reduction in the time of prevalence of CLI pattern after five swimming familiarization sessions within a total of 14 sessions at both moderate and several intensities without to promote physiological adaptation (Lima et al., 2017). Thus, adequate swimming tanks and previous swimming familiarization are the main strategy to reduce CLI pattern occurrence in forced swim tests.

In forced swim tests with weight load or tethered swimming, it is expected a uniform and continuous type of physical activity on the water surface for allowing the animal to breath normally. Therefore, the swimming pattern that best fits this description was classified as CON (Figure 2C). The swimming force analysis suggests that the swimming force for CON pattern was slightly lower than CLI and slightly higher than BOB, although these differences were not statistically significant (Table 3). Moreover, the CON patterns predominate at swimming intensities of physiological stability, but at intensity above MLSS the animal resorts mainly to BOB pattern, which requires lower swimming force, as strategy to save energy and avoid drowning for longer time (Table 3). Maybe, the careful analysis and interpretation of rat swimming pattern may be used to indirectly estimate the exercise intensities of physiological stability and instability.

The diving activity was previously mentioned in a discussion about the task of distinguishing the voluntary dive from the submersion due to exhaustion (Miller and Darrow, 1941). Rats can dive voluntarily by immersing upside down toward the tank bottom, in turn, when they are drowning they sink while struggling to return to the surface. Mainly when the workload to be sustained seems heavy, some rats may dive during several seconds (Figure 2D), apparently with exploratory motivation, seeking to escape from the water tank. Consequently, the apnea due the DIV should have consequences for physiological measurements, as the rise of blood lactate concentration.

Both CLI and DIV patterns are exploratory behaviors because they are more common, in an attempt to escape from water, in naive swimmers (Kregel et al., 2006). Therefore, rats will have to learn after some attempts that no escape route is available. One more time, the swimming familiarization may be a valid strategy to reduce DIV occurrence during forced swim tests. However, some rats still resorted to DIV after 3 weeks of familiarization conducted in the present study. Therefore, specific protocols for inhibit DIV still need to be tested as strategy to reduce DIV occurrence.

Compared to other exercise modalities, the employment of the tethered swimming apparatus enabled an easy and accurate control of the exercise intensity and total amount of work performed in forced swim tests, a feature found previously only in treadmill running. Moreover, the swimming modality can encourage the animal to exercise vigorously without causing mechanical injuries, which is very important in the field of exercise testing and training, while in other modalities, like volunteer well running and forced treadmill running, some rats may refuse to exercise. Aversive stimulus like electroshock can be used to encourage the running exercise but the extent to which the electricity would stress the animals even more remains to be verified. Meanwhile, there is evidence that forced swim is more stressful in rats than treadmill running (Contarteze et al., 2008). Therefore, among other modalities, the forced swim remains as an alternative to promote exercise testing and training in rats.

In the study of Gobatto et al. (2001), the rats were submitted to 20 min of collective swimming exercise, briefly interrupted each 5 min for collecting blood samples. They reported a MLSS-intensity of 5–6% of body mass and a blood lactate concentration of 5.5 mmol.L^-1^. Once the MLSS could be motor pattern dependent (Beneke et al., 2001) and the tethered swimming motor pattern seems to be similar to the swimming with load weight, it was expected similar values of MLSS-intensity and blood lactate concentration between both studies. However, we found lower values for both the MLSS-intensity (∼3.7%BM) and-blood lactate concentration (4.2 mmol.l^-1^) (Tables 2, 3). As reported earlier in humans (Beneke, 2003a,b), the MLSS-intensity and-blood lactate concentration differences between the studies may have occurred due to different test durations and number of interruptions for blood sampling. Additionally, the use of collective swimming by Gobatto et al. (2001) may have changed the exercise intensity variability and motor pattern since the animals are used to hang on each other during the tests (Wilber, 1959; Wilber and Hunn, 1960). Reinforcing the possible protocol dependence of MLSS, a study with procedures more similar to Gobatto et al. (2001) also observed higher values of MLSS workload (4.7% BM) and blood lactate concentration (4.81 mmol/l) than the ones reported here (dos Reis et al., 2011a). Therefore, further studies are necessary to verify the extent to which the number of interruptions for blood sampling, the protocol duration, or even other factors may affect the MLSS-intensity and-blood lactate concentration in rats.

The results suggest that the exercise work accomplished by rats at MLSS-intensity is reproducible when assessed by a tethered-swimming apparatus that considers the SP by the direct determination of SF. However, the BLC is poor correlated in a test–retest in the same condition, probably, due to be affected by other factors besides the exercise intensity. At intensities below and equivalent to MLSS, the CON pattern is the predominant SP, but BOB pattern predominates at intensity above MLSS.

## Author Contributions

IdR, LM, GdA, and CG were involved in the conception and design of the work. IdR carried out the data acquisition, performed the statistical analysis, and wrote the paper. CG made substantial contribution in interpreting data and revising the paper.

## Conflict of Interest Statement

The authors declare that the research was conducted in the absence of any commercial or financial relationships that could be construed as a potential conflict of interest.
